# Delineation of First-Order Elastic Property Closures for Hexagonal Metals Using Fast Fourier Transforms

**DOI:** 10.3390/ma8095303

**Published:** 2015-09-17

**Authors:** Nicholas W. Landry, Marko Knezevic

**Affiliations:** Department of Mechanical Engineering, University of New Hampshire, Durham, NH 03824, USA; E-Mail: nwt8@wildcats.unh.edu

**Keywords:** property closures, elastic properties, orientation distribution function, anisotropy, spectral methods

## Abstract

Property closures are envelopes representing the complete set of theoretically feasible macroscopic property combinations for a given material system. In this paper, we present a computational procedure based on fast Fourier transforms (FFTs) for delineation of elastic property closures for hexagonal close packed (HCP) metals. The procedure consists of building a database of non-zero Fourier transforms for each component of the elastic stiffness tensor, calculating the Fourier transforms of orientation distribution functions (ODFs), and calculating the ODF-to-elastic property bounds in the Fourier space. In earlier studies, HCP closures were computed using the generalized spherical harmonics (GSH) representation and an assumption of orthotropic sample symmetry; here, the FFT approach allowed us to successfully calculate the closures for a range of HCP metals without invoking any sample symmetry assumption. The methodology presented here facilitates for the first time computation of property closures involving normal-shear coupling stiffness coefficients. We found that the representation of these property linkages using FFTs need more terms compared to GSH representations. However, the use of FFT representations reduces the computational time involved in producing the property closures due to the use of fast FFT algorithms. Moreover, FFT algorithms are readily available as opposed to GSH codes.

## 1. Introduction

Property closures are defined as the complete set of the single-crystal and polycrystalline homogenized anisotropic property combinations for a given material. These constructs are important for microstructure based material design. Within the applied mathematics community, the property closures are referred to as the G-closures. In the past, the G-closures have been calculated for properties such as effective conductivity and elastic stiffness for a limited set of two-dimensional microstructures comprised of isotropic phases [1,2,3,4]. More recently, a mathematical framework called microstructure sensitive design (MSD) has been conceived to treat microstructure as a continuous design variable in engineering design and optimization. MSD features invertible or bi-directional linkages between the statistical description of a microstructure and its effective properties [5,6]. These linkages facilitate the identification of microstructures satisfying targeted properties and component performance criteria through the use of the spectral representation of microstructure and material properties. This representation of microstructure facilitates building a space of all theoretically feasible microstructures. When statistical orientation distribution functions (ODFs) are the descriptor of microstructure, the complete set of theoretically feasible ODFs is called a texture hull [5,6,7,8,9] and can be quantitatively described by Fourier coefficients in the multidimensional Fourier space. Mapping the hull into a space of material properties using appropriate ODF-to-property relationships defines the property closures.

In prior work, the spectral representation necessary for MSD was implemented most commonly with generalized spherical harmonics (GSH) basis functions [10]. The GSH bases offer the most compact representations for describing the dependence of anisotropic material properties on crystal orientation because they can be customized to reflect the crystal symmetry as well as the sample symmetry [10,11]. These bases were used to delineate a number of property closures for both cubic and hexagonal metals including combinations of the elastic [7,12,13,14] and plastic [15,16] properties as well as calculations of functional properties [17]. The elastic-plastic property closures presented in these works were based on the first-order bounding theories [18,19,20]. To calculate the bounds of properties, material databases of microstructure invariant Fourier coefficients have to be built. With the databases, calculation of property bounds reduces to multiplication of the spectral coefficients for properties and those for texture (a point in the texture hull) and their summation. These calculations are essentially instantaneous, which is highly desirable for the fast computation of property closures and microstructure based material design. After calculating bounds for selected property combinations of interest by mapping the hull into property space, the problem at hand is to find textures corresponding to the boundary points of the property closure. To this end, several computational methodologies were formulated [5,12,21,22]. In the core of these methodologies is an appropriately formulated optimization procedure aimed at finding textures corresponding to the boundary points of the property closure. Interestingly, for all cubic metals, textures at the boundary points of the property closures correspond to the same set of textures [13].

Several examples exist in the current literature on using property closures to theoretically design and optimize microstructure to improve performances of components. These examples identified microstructures and associated properties maximizing the deflection in a compliant beam [5], the energy storage of a flywheel [23], and the in-plane load carrying capacity of a thin plate with a central circular hole [6] and minimizing the elastic driving force for crack extension in rotating disks [24] and internally pressurized thin-walled vessels [25]. The main microstructural feature governing the elastic and plastic properties in these studies has been the ODF, although in MSD, some limited consideration has been given to compositional variations in microstructures [26] and to fiber reinforced composites [9,27]. A design strategy termed the topology optimization has been successful in designing composites of extreme functional and mechanical properties [28,29,30,31].

In crystal plasticity [32,33,34,35,36,37,38,39,40,41,42,43,44,45,46,47,48], calculations of average Taylor factors and Mandel spins for all possible strain modes have also been accomplished using the GSH bases [49]. In addition, the GSH bases were used to build databases of precomputed crystal plasticity solutions for the stress, strain hardening and texture evolution function for cubic metals [50,51]. Evaluation of these functions reduced to multiplying the orientation invariant spectral coefficient with the orientation dependent GSH basis and summing as opposed to iteratively solving them using Newton’s solvers was found to accelerate crystal plasticity models. Recently, in place of the GSH representations, fast Fourier Transforms (FFTs) have been used in the ODF representation [52] as well as the crystal plasticity framework [16,53,54,55,56,57,58]. The main motivation for using FFTs is the availability of much more computationally efficient algorithms than those using GSH coefficients. The methods based on GSH are significantly less effective when applied to lower symmetry metals because the relevant number of dimensions in the Fourier space is considerably higher than that for cubic metals [7]. Additionally, FFT libraries are readily accessible while GSH codes are not as easily found. Delineation of property closures for cubic metals using FFTs has recently been accomplished [52].

In this paper, we have successfully formulated a new approach using FFTs to compute elastic property closures for HCP polycrystals. To this end, we have built a database of the orientation invariant Fourier transforms for components of the elastic stiffness and developed an optimization scheme for finding the boundary points of the convex property envelopes. This new approach is introduced in this paper and demonstrated with examples. The selected materials for this study were titanium, zinc, zirconium, beryllium, magnesium, and cobalt. These materials were chosen to validate the FFT based closures against reported property closures based on the GSH representation for orthotropic HCP metals [7]. It was shown that the closures obtained using the FFT representation are identical to those based on the GSH representation for the orthotropic terms of the stiffness tensor. The FFT representation allows delineation of first-order closures of elastic properties involving the normal–shear coupling stiffness/compliance tensor components. Although polycrystalline elastic properties were calculated from a given texture in the most general formalism of triclinic sample symmetry using the Voigt-Reuss-Hill approximation [11], this is the first report of this type of closures for HCP metals. Finally, we have shown that it is possible to build texture hulls using FFTs for HCP polycrystals.

## 2. Representation of ODF Using FFTs and Texture Hulls for HCP Metals

The Orientation Distribution Function (ODF), f(g), is the probability density associated with the occurrence of the crystallographic orientation, *g*, in the sample of a polycrystalline material. An ODF can be mathematically expressed in its continuous or discrete form. While the former is required for the GSH representation, the latter is necessary for the FFT representation. In its discrete form, an ODF is expressed as [59]:

(1) $$f_{b}\Delta g=\frac{N_{g\pm \Delta g/2}}{N},\;\sum_{b\in FZ}f_{b}\Delta g=1$$

where *N* is the total number of orientations in the sample; $N_{g\;\pm \;\Delta g/2}$ is the number of orientations that lie within a bin Δg centered about *g*. FZ and *b* will be defined shortly. The orientation, *g*, can be described using an ordered set of three Bunge-Euler rotation angles $\left(\phi_{1},\text{Φ},\phi_{2}\right)$ that bring into coincidence the crystal axis with the sample reference frame. The Bunge-Euler representation was employed because of the inherent periodicity of the space when defined as $\left(\phi_{1}\in \left[0,2\text{π}\right),\text{ Φ}\in \left[0,2\text{π}\right),\phi_{2}\in \left[0,2\text{π}\right)\right)$. On this domain, any function of crystal orientations is guaranteed to be periodic and circumvent the Gibbs phenomenon [60], eliminating the need for additional high frequency terms in the FFT representations. The Bunge-Euler space is defined by the rotation angles that make their appearance in structure-property relationships of interest in the form of integer powers of sines and cosines of the rotation angles. The main disadvantages of the Bunge-Euler space are the numerous redundancies in the representation of crystal orientations (*i.e.*, several locations in this space may correspond to a single crystal orientation) and the inherent distortion caused by the fact that the invariant measure in this space is proportional to $\Delta g=sin\text{ΦΔ}\phi_{1}\text{ΔΦΔ}\phi_{2}$ [10]. In spite of the disadvantages of the Bunge-Euler space, the development of the spectral representations has been most successful for this space, largely because of the implicit periodicity in the material property functions of interest when defined in this space.

Knowing the function values on a uniform grid, FFTs can be computed [61,62,63,64,65]. In order to represent the ODF using FFTs, we sample the Bunge-Euler space using this grid, which defines the bins. The FFT representation of an ODF is expressed as:

(2) $$f_{b_{1,}b_{2},b_{3}}=\frac{1}{B_{1}B_{2}B_{3}}\overset{B_{1}-1}{\underset{k_{1}=0}{\sum }}\overset{B_{2}-1}{\underset{k_{2}=0}{\sum }}\overset{B_{3}-1}{\underset{k_{3}=0}{\sum }}F_{k_{1}k_{2}k_{3}}e^{\frac{2\text{π}ib_{1}k_{1}}{B_{1}}}e^{\frac{2\text{π}ib_{2}k_{2}}{B_{2}}}e^{\frac{2\text{π}ib_{3}k_{3}}{B_{3}}}$$

where $f_{b_{1,}b_{2},b_{3}}$ denotes the value of the ODF at the grid point identified by *b*, and $b_{1}$, $b_{2}$, and $b_{3}$ enumerate points within uniformly discretized domain; $F_{k_{1}k_{2}k_{3}}$ are the FFTs of the ODF indexed by *k*, while $k_{1}$, $k_{2}$, and $k_{3}$ are the indices of the FFTs; and $B_{1}$, $B_{2}$, and $B_{3}$ are the total number of grid points in the periodic domain. Since the values of the ODF are the real half of the transforms, the other half are complex conjugates *i.e.*, $F_{k_{1},k_{2},k_{3}}=F_{B_{1}-k_{1},B_{2}-k_{2},B_{3}-k_{3}}^{*}$. The superscript, ^*^, denotes the complex conjugate transforms.

For simplicity of notation, Equation (2) will be expressed in a shortened notation as:

(3) $$f_{b}=\frac{1}{B}\overset{B-1}{\underset{k=0}{\sum }}F_{k}e^{\frac{2\text{π}ibk}{B}}.$$

The shortened notation will be used hereafter. For given values of the ODF on the grid over *B*, the FFTs are:

(4) $$F_{k}=\overset{B-1}{\underset{k=0}{\sum }}f_{b}e^{-\frac{2\text{π}ibk}{B}.}$$

Computing the ODF transforms using the FFT methods was over two orders of magnitude faster compared to that obtained using the GSH methods. Benefits of the FFT method over the GSH method increase with the number of ODFs considered in the calculations of property closures because the computational time involved in ODF-to-elastic property calculations scales linearly with the number of ODFs.

Due to hexagonal crystal symmetry, there are 24 physically indistinctive orientations over the discretized Bunge-Euler space defined above, *B*. *FZ* is the fundamental zone within *B* containing the complete set of all physically distinct orientations that can occur in the sample. These redundancies within *B* can be exploited in the computations. To this end, we calculate function values in *FZ* and translate these throughout the rest of the space *B* while preserving the uniform grid. The translations are done as follows. For crystals of any symmetry, the definitions of the Bunge-Euler angles require that locations $\left(\phi_{1}+\text{π},\;2\text{π}-\text{Φ},\;\phi_{2}+\text{π}\right)$ correspond to the exact same crystal lattice orientation. Table 1 shows twelve equivalent crystal orientations within the rotational point symmetry group associated with HCP crystals.

**Table 1:** Twelve symmetry operations within hexagonal close packed (HCP) rotational point symmetry group.

Symmetries 1–4	Symmetries 5–8	Symmetries 9–12
$\left(\phi_{1},\text{Φ},\phi_{2}\right)$	$\left(\phi_{1},\text{Φ},\phi_{2}+\frac{\text{π}}{3}\right)$	$\left(\phi_{1},\text{Φ},\phi_{2}+\frac{2\text{π}}{3}\right)$
$\left(\phi_{1},\text{Φ},\phi_{2}+\text{π}\right)$	$\left(\phi_{1},\text{Φ},\phi_{2}+\frac{4\text{π}}{3}\right)$	$\left(\phi_{1},\text{Φ},\phi_{2}+\frac{5\text{π}}{3}\right)$
$\left(\phi_{1},\text{Φ}+\text{π},\phi_{2}\right)$	$\left(\phi_{1},\text{Φ}+\text{π},\phi_{2}+\frac{\text{π}}{3}\right)$	$\left(\phi_{1},\text{Φ}+\text{π},\phi_{2}+\frac{2\text{π}}{3}\right)$
$\left(\phi_{1},\text{Φ}+\text{π},\phi_{2}+\text{π}\right)$	$\left(\phi_{1},\text{Φ}+\text{π},\phi_{2}+\frac{4\text{π}}{3}\right)$	$\left(\phi_{1},\text{Φ}+\text{π},\phi_{2}+\frac{5\text{π}}{3}\right)$

As a result of all of the considerations described above, the *FZ* space for HCP is $FZ=\left\{g=\left(\phi_{1},\text{Φ},\phi_{2}\right)\;|\;0\leq \phi_{1}\leq 2\text{π},0\leq \text{Φ}\leq \frac{\text{π}}{2},0\leq \phi_{2}\leq \frac{\text{π}}{3}\right\}$. Values of any desired function can be calculated on a uniform grid in *FZ* and subsequently extended over the entire periodic space using the above defined equivalencies preserving the uniform grid over *B*.

It should be noted that the smallest space defined earlier for cubic metals was defined as *FZ3* [52], which is three times the actual *FZ* for cubic crystals. The reason for using *FZ3* instead of *FZ* is that a uniform grid is only possible in *FZ3*. *FZ* upon being subjected to three-fold <111> symmetry operation, does not result in a uniform grid that can be used to fill out the remainder of the *B* space [8]. Therefore, the values of all desired functions on a uniform grid span the *FZ3* space for cubic crystals while they conveniently span the *FZ* domain for HCP crystals.

The FFT representation allows defining the texture hull for HCP crystals. Let $F_{k}^{n}$ define the FFTs of single crystals that lie within *FZ*, which are computed using Equation (4). Let *N* enumerate the single crystals that are distributed over in *FZ*. It is then possible to define a convex and compact texture hull [5] as:

(5) $$M=\left\{F_{k}\;|\;F_{k}=\overset{N}{\underset{n=1}{\sum }}\alpha_{n}F_{k}^{n},\;\alpha_{n}\geq 0,\;\overset{N}{\underset{n=1}{\sum }}\alpha_{n}=1\right\}$$

**Figure 1:**
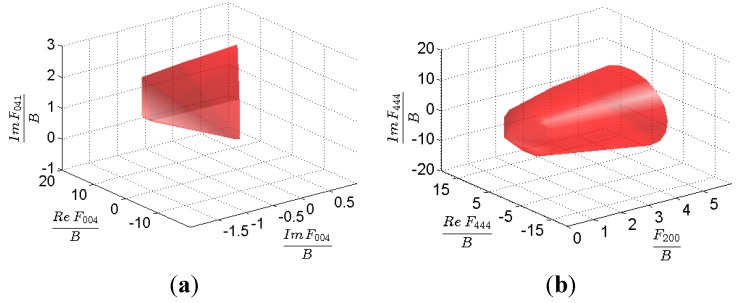
Examples of the fast Fourier transforms (FFT) texture hull for HCP-triclinic materials. Two arbitrarily selected normalized projections are: (**a**) *ReF_004_*, *ImF_004_*, *ImF_041_* and (**b**) *ReF_444_*, *F_200_*, *ImF_444_*.

As mentioned earlier, *M* represents the complete set of all theoretically feasible ODFs including all of their linear combinations. *M* is defined in n-dimensional FFT space, where the number of dimensions in this space is equal to the discretization *B*. Selected projections of *M* are presented in Figure 1. The FFT based hulls are analogous to those presented previously using the GSH representations [7]. In the next section, we describe the methodology for mapping the texture hull in the material property space and construction of HCP property closures.

## 3. Property Closures

In previous work [7], it was demonstrated that a spectral approach based on the GSH basis can be used to construct property closures for HCP metals. Here, we show that the elastic property closures can be computed much more efficiently using the more readily accessible FFT representation. The primary focus here continues to be on the crystallographic texture in the sample as primary description of the microstructure. All of the closures reported in prior studies on HCP employed the orthotropic GSH functions, which implies orthotropic sample symmetry. The closures presented here using the FFT representations circumvent this symmetry assumption.

### 3.1. Elastic Stiffness for HCP Metals

The components of the stiffness tensor for an HCP single crystal as a function of the local crystal orientation and the five fundamental elastic constants $C_{11},\;C_{12},C_{13},C_{33},$ and $C_{44}$ expressed in the sample frame are:

(6) $$\begin{array}{ll} C_{abcd}= & C_{12}{\text{δ}}_{ab}{\text{δ}}_{cd}+C_{44}({\text{δ}}_{ad}{\text{δ}}_{bc}+{\text{δ}}_{ac}{\text{δ}}_{bd})\\ & \;+(C_{11}-C_{12}-2C_{44})\;(\overset{3}{\underset{t=1}{\sum }}g_{at}g_{bt}g_{ct}g_{dt}\\ & \;+\frac{1}{2}(g_{a1}g_{b2}g_{c1}g_{d2}+g_{a1}g_{b2}g_{c2}g_{d1}+g_{a2}g_{b1}g_{c1}g_{d2}+g_{a2}g_{b1}g_{c2}g_{d1}))\\ & \;+(C_{13}-C_{12})\overset{2}{\underset{t=1}{\sum }}(g_{at}g_{bt}g_{c3}g_{d3}+g_{a3}g_{b3}g_{ct}g_{dt})\\ & +(C_{33}-C_{11})g_{a3}g_{b3}g_{c3}g_{d3} \end{array}$$

where ${\text{δ}}_{ab}$ represents the Kronecker symbol and $g_{ab}$ is:

(7) $$g_{ab}=[\begin{array}{ccc} \cos\phi_{1}\cos\phi_{2}-\sin\phi_{1}\cos\text{Φ}\sin\phi_{2} & -\cos\phi_{1}\sin\phi_{2}-\sin\phi_{1}\cos\text{Φ}\sin\phi_{2} & \sin\phi_{1}\sin\text{Φ}\\ \sin\phi_{1}\cos\phi_{2}+\cos\phi_{1}\cos\text{Φ}\sin\phi_{2} & -\sin\phi_{1}\sin\phi_{2}+\cos\phi_{1}\cos\text{Φ}\cos\phi_{2} & -\cos\phi_{1}\sin\text{Φ}\\ \sin\text{Φ}\sin\phi_{2} & \sin\text{Φ}\cos\phi_{2} & \cos\text{Φ} \end{array}]$$

We use bold letters to denote tensors. It is convenient to separate the orientation dependence of the tensor [7] as:

(8a) $$\begin{array}{c} A\left(g\right)=A_{abcd}\left(g\right)=\sum_{t=1}^{3}g_{at}g_{bt}g_{ct}g_{dt}+\frac{1}{2}(g_{a1}g_{b2}g_{c1}g_{d2}\\ +g_{a1}g_{b2}g_{c2}g_{d1}+g_{a2}g_{b1}g_{c1}g_{d2}+g_{a2}g_{b1}g_{c2}g_{d1}) \end{array}$$

(8b) $$B\left(g\right)=B_{abcd}\left(g\right)=\sum_{t=1}^{2}(g_{at}g_{bt}g_{c3}g_{d3}+g_{a3}g_{b3}g_{ct}g_{dt})$$

(8c) $$D\left(g\right)=D_{abcd}\left(g\right)=\;g_{a3}g_{b3}g_{c3}g_{d3}$$

Equation (6) then becomes:

(9) $$C=C_{12}{\text{δ}}_{ab}{\text{δ}}_{cd}+C_{44}\left({\text{δ}}_{ad}{\text{δ}}_{bc}+{\text{δ}}_{ac}{\text{δ}}_{bd}\right)+\left(C_{11}-C_{12}-2C_{44}\right)A\left(g\right)\;+\left(C_{13}-C_{12}\right)B\left(g\right)\;+\left(C_{33}-C_{11}\right)D\left(g\right)$$

### 3.2. Representation of the Elastic Stiffness for HCP Metals Using FFTs

Orientation dependent functions $A_{abcd}\left(g\right)$, $B_{abcd}\left(g\right)$, and $D_{abcd}\left(g\right)$ can be evaluated on a uniform grid over *B* and represented using FFTs as:

(10) $${\tilde{P}}_{k}^{\text{A}}=\sum_{k=0}^{B-1}A_{b}sin\text{Φ}e^{-\frac{2\text{π}ibk}{B}},{\tilde{P}}_{k}^{\text{B}}=\sum_{k=0}^{B-1}B_{b}sin\text{Φ}e^{-\frac{2\text{π}ibk}{B}},{\tilde{P}}_{k}^{\text{D}}=\sum_{k=0}^{B-1}D_{b}sin\text{Φ}e^{-\frac{2\text{π}ibk}{B}}$$

To evaluate the property transforms, we had to evaluate the functions (Equation (8)) on a regular grid over *B*. The grid was refined to capture all of the non-zero frequencies present in the functions. A grid with one degree grid point spacing placed over *B* was found to satisfy this requirement, meaning that all of the important frequencies were captured by the transform. The non-zero Fourier transforms for elastic stiffness components are presented in Appendix A. Note that these transforms are orientation invariant and valid for all HCP metals. The database contains only half of the transforms since ${\tilde{P}}_{k}^{\text{A}}={\tilde{P}*}_{B-k}^{\text{A}},{\tilde{P}}_{k}^{\text{B}}={\tilde{P}*}_{B-k}^{\text{B}},\;\text{and}\;{\tilde{P}}_{k}^{\text{D}}={\tilde{P}*}_{B-k}^{\text{D}}$. Note that for example ${\tilde{P}}_{k}^{\text{A}}$ is a compact way of writing P˜kAabcd=P˜kA. The tilde indicates that the functions have been pre multiplied by sinΦ term. The term will be clarified shortly. As can be seen in the database, the number of transforms varies for the different stiffness components. Comparing the GSH Fourier coefficients presented in [7] for the same functions, we note that the FFT representation has a few more terms but the FFTs are tremendously faster and readily available.

### 3.3. First-Order Elastic Stiffness Bounds

The FFT representations in Equation (10) permit the efficient computation of the property bounds for any given ODF from the HCP texture hull. The first-order upper and lower bounds of effective elastic stiffness for the diagonal components are expressed as [12,18,19,20]:

(11) $${\left({\bar{S}}^{-1}\right)}_{abab}\leq C_{abab}^{*}\leq {\bar{C}}_{abab}$$

and for the off-diagonal components, the bounds are expressed as:

(12a) $$\begin{array}{ll} \max({\bar{C}}_{abcd}, & {\left({\bar{S}}^{-1}\right)}_{abcd})-\sqrt{{\text{Δ}}_{abab}{\text{Δ}}_{cdcd}}\leq C_{abcd}^{*}\\ & \leq \min({\bar{C}}_{abcd},{\left({\bar{S}}^{-1}\right)}_{abcd})+\sqrt{{\text{Δ}}_{abab}{\text{Δ}}_{cdcd}} \end{array}$$

(12b) $${\text{Δ}}_{abcd}={\bar{C}}_{abcd}-{\left({\bar{S}}^{-1}\right)}_{abcd}$$

In Equations (11) and (12), no implicit summation on repeated indices is used (The Einstein indicial notation of implicit summation on repeated indices is employed in this paper, except when explicitly noted otherwise). The bars on top of a field quantity denote the volume averaged value over constituent crystals in a polycrystal: ${\left({\bar{S}}^{-1}\right)}_{abab}$ is the polycrystal compliance matrix with an inverse taken after homogenization and ${\bar{C}}_{abab}$ is the polycrystal stiffness matrix. This paper is the first work to compute off-diagonal term HCP property closures.

### 3.4. Homogenization of the Elastic Properties in Fourier Space

As mentioned in the previous section, the first-order homogenization theory requires calculations of the volume average quantities. As an example, the upper bound for the components of the homogenized elastic stiffness tensor can be expressed as the following (no implicit summation on repeated indices):

(13) $$\bar{C}={\bar{C}}_{abcd}=\frac{1}{V}\int_{V}C_{abcd}\left(x\right)dx=\int_{FZ3}f\left(g\right)C_{abcd}\left(g\right)sin\text{Φ}d\phi_{1}d\text{Φ}d\phi_{2}=C_{12}\delta_{ab}\delta_{cd}+\;C_{44}\left(\delta_{ad}\delta_{bc}+\delta_{ac}\delta_{bd}\right)+\frac{1}{B}\sum_{k=0}^{B-1}[\left(C_{11}-C_{12}-2C_{44}\right){\tilde{P}}_{k}^{A}+\left(C_{13}-C_{12}\right){\tilde{P}}_{k}^{B}+\;(C_{33}-C_{11}){\tilde{P}}_{k}^{D}]F_{k}$$

where *V* is the physical volume of a sample and **x** is a location in the sample. Alternatively, the volume average functions **A**, **B** and **D** can be calculated by multiplication of the precomputed ${\tilde{P}}_{k}^{\text{A}}$, ${\tilde{P}}_{k}^{\text{B}}$, and ${\tilde{P}}_{k}^{\text{D}}$ with the ODF transforms *F_k_* and their summation as: ${\bar{A}}_{abcd}=\frac{1}{B}\overset{B-1}{\underset{k=0}{\sum }}{\tilde{P}}_{k}^{\text{A}}F_{k}$, ${\bar{B}}_{abcd}=\frac{1}{B}\overset{B-1}{\underset{k=0}{\sum }}{\tilde{P}}_{k}^{\text{B}}F_{k}$, ${\bar{D}}_{abcd}=\frac{1}{B}\overset{B-1}{\underset{k=0}{\sum }}{\tilde{P}}_{k}^{\text{D}}F_{k}$. These quantities can be used in Equation (9) to calculate the homogenized elastic stiffness tensor. Similar expressions exist for all tensors involved in Equations (11) and (12). Note that we have exploited the orthogonal properties of the spectral representation in the last part of the Equation (13). Since the regular grid is used in the summations in each direction incrementally $\left(\Delta \phi_{1},\Delta \text{Φ},\Delta \phi_{2}\right)$, the sinΦ accounting for the distortion of the Bunge-Euler space must multiply either f(g) or $C_{abcd}\left(g\right)$ (Equation (13)). We have chosen to multiply the functions **A**, **B** and **D** by the sinΦ term, therefore we have added tilde symbol to the property transforms.

**Figure 2:**
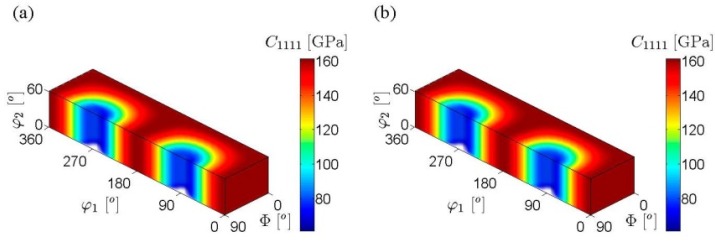
Contour plots of the $C_{1111}$ elastic stiffness component in the HCP fundamental zone of the Bunge-Euler space for Zn: (**a**) computed using the non-zero FFTs (Equation (13)) and (**b**) computed directly using Equation (6). The maximum difference between corresponding locations in the two plots is of the order 10^−6^ GPa.

Equation (13) can be used to calculate the volume average elastic stiffness as well as the elastic stiffness of a single crystal. It should be noted that the number of calculations (multiplications and summations) for stiffness components is the same for a single crystal and a polycrystal comprised of any number of crystals in this spectral representation. The number of calculations is determined by the number of non-zero transforms (Appendix A). Figure 2 shows contour plots of the $C_{1111}\left(g\right)$ elastic stiffness component over *FZ* for HCP metals calculated using Equations (6) and (13). The single crystal elastic constants of Zn were used. It can be clearly seen that the results are identical, which validates the FFT representation of the ODF-to-elastic stiffness relationship. The same checks were conducted for all components of the local elastic stiffness tensor.

### 3.5. Computation of Property Closures for HCP Metals

The methodology used here for building first-order elastic stiffness closures starts with a consideration of a set of points in the texture hull that correspond to “eigen textures” [66]. The set of eigen textures is selected while ensuring an adequate coverage of the HCP FZ. The property bounds (Equations (11) and (12)) are first evaluated using the FFT representation of the ODF-to-elastic stiffness linkages (Equation (13)) for these eigen textures. A finite number of eigen textures corresponding to the boundary of a given property closure of stiffness components was then selected. Subsequently, the property combinations were evaluated for weighted combinations of these textures, taking one pair of textures at a time. The weighted combinations of pairs of the selected textures were incremented by a 0.1 weighted fraction *i.e.*, from (0.1, 0.9) pair to (0.9, 0.1) pair, which resulted with nine weighted combinations. As expected, these computations involving combinations of textures expand the property closures. Next, a new set of textures corresponding to the new boundary of the expanded closure were selected (this time these were a mixture of eigen textures and non-eigen textures) and the property combinations corresponding to the weighted combinations of these were evaluated. This process was repeated until the closure expansion saturated. The method of delineation of closures follows the main ideas underlying genetic algorithms, where good solutions are pre-selected and used in further calculations. This approach was applied successfully to delineate closures of plastic properties involving uniform ductility and ultimate tensile strength in cubic metals in [15].

### 3.6. Atlases of Property Closures for HCP Metals

**Figure 3:**
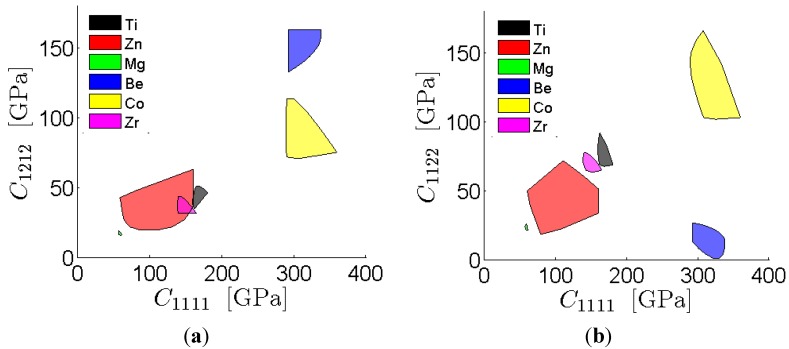
Atlases of property closures for a range of HCP metals computed using the FFT method: (**a**) *C*_1111_ *vs.* *C*_1212_ and (**b**) *C*_1111_ *vs.* *C*_1122_.

**Figure 4:**
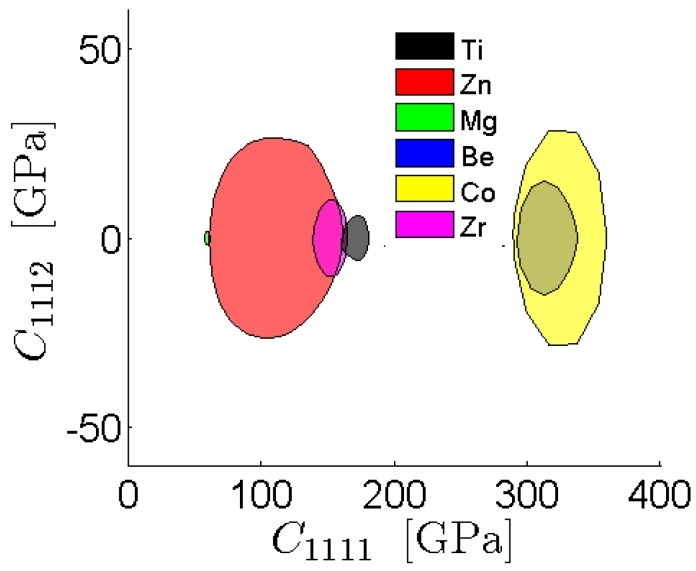
Atlases of the normal-shear coupling stiffness closures for a range of HCP metals computed using the FFT method.

The FFT representation of the ODF-to-elastic stiffness bounds is used in this study to obtain the first-order property closures, which identify the complete set of theoretically realizable combinations of selected macroscale properties in a given material system through a consideration of the complete set of textures (*i.e.*, the texture hull). The shaded areas inside the closures represent the possible property combinations of the selected elastic stiffness components that can be obtained according to the first-order bounding theories for the particular material as a function of ODFs. We provide three examples of atlases of property closures corresponding to selection of different pairs of effective elastic properties. Figure 3 depicts examples of the property closures produced in this work using the FFT representations for a range of HCP metals. This particular property combination of the effective modulus in unaxial strain and the effective shear modulus in the sample play an important role in the design of components subjected simultaneously to axial loads and twisting moments. The single crystal elastic constants for these metals were taken from literature [67]. Comparison with the closures previously reported in [7], which were computed using the GSH representations reveals excellent agreement. It should be noted that this agreement occurs in spite of the fact that the previously computed closures assumed orthotropic sample symmetry, whereas the computations reported here did not invoke any sample symmetry assumption. This is because the elastic stiffness components involved in these two closures exhibit orthotropic sample symmetry. Figure 4 depicts another set of closures for the same set of metals that involve the coupling of the normal-shear components. When orthotropic sample symmetry is assumed, $C_{1112}$ is zero. The closures presented here in Figure 4 show that the values of $C_{1112}$ can take a wide range, which can play an important role in the macroscale mechanical response of components (note that these are often ignored in practice by assuming an orthotropic sample symmetry that the material may not exhibit).

As a final comment, we note that the FFT framework developed here for the efficient calculation of elastic properties can also be used to accelerate the calculations of the elasto-viscoplastic response of polycrystalline HCP metals that is under development. This framework has been recently reported for cubic metals in [68]. The implementation presented in [68] takes the advantage of calculations of the elastic properties based on the spectral representation reported in [52]. For HCP, the components of the local elastic stiffness tensor as a function of the crystal orientation can be calculated using Equation (13) instead of Equation (6). Therefore, the elastic stiffness solutions obtained based on the FFT representation can be used within the elasto-viscoplastic crystal plasticity constitutive laws operating at every integration point within implicit finite elements [69,70,71,72,73,74,75].

## 4. Conclusions

In this paper, we have extended the recently developed first-order ODF-to-elastic stiffness linkages using FFTs for cubic metals to more computationally challenging HCP polycrystals. The benefit of using FFTs to represent these linkages is the substantial reduction in the computational time involved in the property calculations due to the use of fast and readily available FFT algorithms. First, we have shown that it is possible to construct texture hulls using FFTs for HCP polycrystals, as was done earlier for cubic polycrystals. Next, we have calculated elastic stiffness FFTs and presented the actual values in Appendix A. Consistent with the GSH representation, we found that the inherently more anisotropic HCP structure demands a larger number of FFTs than FFTs used for the cubic structure to represent these properties. However, the use of FFT representations reduced the computational time involved in producing the property closures due to the use of efficient FFT algorithms. Finally, we have successfully computed elastic property closures for HCP metals without invoking any sample symmetry assumption. To this end, the methodology presented here facilitated for the first time the delineation of the property closures involving the normal–shear coupling stiffness coefficients.

## Appendix A

**Table:** ${\tilde{P}}_{k_{1},k_{2},k_{3}}^{\text{A}}$

*k*_1_ *k*_2_ *k*_3_	*C*_1111_	*k*_1_ *k*_2_ *k*_3_	*C*_1112_	*k*_1_ *k*_2_ *k*_3_	*C*_1113_
0 0 0	3.645 × 10^7^ + 0j	0 0 0	1.346 × 10^−3^ + 0j	0 0 0	3.474 × 10^−4^ + 0j
0 0 2	2.916 × 10^6^ − 8.017 × 10^−4^j	0 0 2	−1.024 × 10^−3^ − 1.458 × 10^6^j	0 2 1	1.458 × 10^6^ − 1.752 × 10^−3^j
0 0 4	2.187 × 10^6^ + 1.490 × 10^−3^j	0 0 4	−3.287 × 10^−4^ − 2.187 × 10^6^j	0 2 3	1.458 × 10^6^ − 9.201 × 10^−4^j
0 2 0	2.916 × 10^6^ − 8.017 × 10^−4^j	0 2 4	5.437 × 10^−4^ + 1.458 × 10^6^j	0 2 357	−1.458 × 10^6^ + 8.970 × 10^−4^j
0 2 4	−1.458 × 10^6^ − 2.353 × 10^−4^j	0 2 356	3.103 × 10^−4^ − 1.458 × 10^6^j	0 2 359	−1.458 × 10^6^ + 1.470 × 10^−3^j
0 2 356	−1.458 × 10^6^ − 1.324 × 10^−3^j	0 4 2	5.819 × 10^−4^ + 7.290 × 10^5^j	0 4 1	2.187 × 10^6^ − 3.252 × 10^−3^j
0 4 0	2.187 × 10^6^ + 1.490 × 10^−3^j	0 4 4	−1.197 × 10^−3^ − 3.645 × 10^5^j	0 4 3	−7.290 × 10^5^ + 7.335 × 10^−4^j
0 4 2	−1.458 × 10^6^ − 2.353 × 10^−4^j	0 4 356	−3.065 × 10^−4^ + 3.645 × 10^5^j	0 4 357	7.290 × 10^5^ + 1.229 × 10^−3^j
0 4 4	3.645 × 10^5^ − 4.225 × 10^−4^j	0 4 358	−1.105 × 10^−3^ − 7.290 × 10^5^j	0 4 359	−2.187 × 10^6^ + 1.318 × 10^−3^j
0 4 356	3.645 × 10^5^ − 2.071 × 10^−12^j	–	–	–	–
0 4 358	−1.458 × 10^6^ + 1.324 × 10^−3^j	–	–	–	–
*k*_1_ *k*_2_ *k*_3_	*C*_1122_	*k*_1_ *k*_2_ *k*_3_	*C*_1123_	*k*_1_ *k*_2_ *k*_3_	*C*_1133_
0 0 0	4.374 × 10^6^ + 0j	0 0 0	−8.664 × 10^−4^ + 0j	0 0 0	5.832 × 10^6^ + 0j
0 0 4	−2.187 × 10^6^ + 7.111 × 10^−4^j	0 2 1	−3.505 × 10^−3^ + 1.458 × 10^6^j	0 0 2	−2.916 × 10^6^ + 1.366 × 10^−5^j
0 2 0	−2.916 × 10^6^ + 3.868 × 10^−3j^	0 2 3	2.036 × 10^−3^ − 1.458 × 10^6^j	0 4 0	−2.916 × 10^6^ + 3.465 × 10^−3^j
0 2 4	1.458 × 10^6^ − 2.976 × 10^−3^j	0 2 357	−1.196 × 10^−3^ − 1.458 × 10^6^j	0 4 2	1.458 × 10^6^ − 2.873 × 10^−3^j
0 2 356	1.458 × 10^6^ − 2.763 × 10^−3^j	0 2 359	2.458 × 10^−3^ + 1.458 × 10^6^j	0 4 358	1.458 × 10^6^ − 2.842 × 10^−3^j
0 4 0	7.290 × 10^5^ − 4.308 × 10^−3^j	0 4 1	4.336 × 10^−3^ − 7.290 × 10^5^j	–	–
0 4 4	−3.645 × 10^5^ + 2.327 × 10^−3^j	0 4 3	−3.380 × 10^−3^ + 7.290 × 10^5^j	–	–
0 4 356	−3.645 × 10^5^ + 3.997 × 10^−3^j	0 4 357	2.269 × 10^−4^ + 7.290 × 10^5^j	–	–
–	–	0 4 359	−2.354 × 10^−3^ − 7.290 × 10^5^j	–	–
*k*_1_ *k*_2_ *k*_3_	*C*_1212_	*k*_1_ *k*_2_ *k*_3_	*C*_1312_	*k*_1_ *k*_2_ *k*_3_	*C*_1313_
0 0 0	1.604 × 10^7^ + 0j	0 0 0	5.001 × 10^−4^ + 0j	0 0 0	1.166 × 10^7^ + 0j
0 0 4	−2.187 × 10^6^ + 1.968 × 10^−3^j	0 2 1	−2.863 × 10^−3^ − 1.458 × 10^6^j	0 2 0	−2.916 × 10^6^ + 1.449 × 10^−3^j
0 2 0	2.916 × 10^6^ − 3.666 × 10^−4^j	0 2 3	1.106 × 10^−4^ − 1.458 × 10^6^j	0 2 2	−1.458 × 10^6^ − 2.397 × 10^−3^j
0 2 4	1.458 × 10^6^ − 3.097 × 10^−3^j	0 2 357	−1.002 × 10^−3^ − 1.458 × 10^6^j	0 2 358	−1.458 × 10^6^ − 2.518 × 10^−3^j
0 2 356	1.458 × 10^6^ − 2.027 × 10^−3^j	0 2 359	−1.417 × 10^−3^ − 1.458 × 10^6^j	0 4 0	−2.916 × 10^6^ + 2.298 × 10^−4^j
0 4 0	7.290 × 10^5^ + 3.823 × 10^−3^j	0 4 1	−7.437 × 10^−4^ − 7.290 × 10^5^j	0 4 2	1.458 × 10^6^ + 1.402 × 10^−3^j
0 4 4	−3.645 × 10^5^ + 1.073 × 10^−3^j	0 4 3	−6.925 × 10^−4^ + 7.290 × 10^5^j	0 4 358	1.458 × 10^6^ + 5.424 × 10^−4^j
0 4 356	−3.645 × 10^5^ + 3.916 × 10^−3^j	0 4 357	−3.324 × 10^−4^ + 7.290 × 10^5^j	–	–
–	–	0 4 359	−8.323 × 10^−5^ − 7.290 × 10^5^j	–	–
*k*_1_ *k*_2_ *k*_3_	*C*_2212_	*k*_1_ *k*_2_ *k*_3_	*C*_2213_	*k*_1_ *k*_2_ *k*_3_	*C*_2222_
0 0 0	−2.227 × 10^−3^ + 0j	0 0 0	1.855 × 10^−3^ + 0j	0 0 0	3.645 × 10^7^ + 0j
0 0 2	−8.014 × 10^−4^ − 1.458 × 10^6^j	0 2 1	−1.458 × 10^6^ + 2.242 × 10^−3^j	0 0 2	−2.916 × 10^6^ + 2.652 × 10^−3^j
0 0 4	1.646 × 10^−3^ + 2.187 × 10^6^j	0 2 3	−1.458 × 10^6^ − 9.985 × 10^−4^j	0 0 4	2.187 × 10^6^ − 2.554 × 10^−3^j
0 2 4	−1.432 × 10^−3^ − 1.458 × 10^6^j	0 2 357	1.458 × 10^6^ − 9.966 × 10^−4^j	0 2 0	2.916 × 10^6^ + 1.752 × 10^−3^j
0 2 356	−1.473 × 10^−3^ + 1.458 × 10^6^j	0 2 359	1.458 × 10^6^ + 2.525 × 10^−3^j	0 2 4	−1.458 × 10^6^ − 8.290 × 10^−5^j
0 4 2	1.079 × 10^−4^ + 7.290 × 10^5^j	0 4 1	7.290 × 10^5^ − 9.776 × 10^−4^j	0 2 356	−1.458 × 10^6^ − 3.054 × 10^−3^j
0 4 4	1.413 × 10^−3^ + 3.645 × 10^5^j	0 4 3	7.290 × 10^5^ + 4.229 × 10^−3^j	0 4 0	2.187 × 10^6^ − 7.525 × 10^−4^j
0 4 356	2.592 × 10^−4^ − 3.645 × 10^5^j	0 4 357	−7.290 × 10^5^ − 1.011 × 10^−4^j	0 4 2	1.458 × 10^6^ − 2.064 × 10^−3^j
0 4 358	−4.406 × 10^−4^ − 7.290 × 10^5^j	0 4 359	−7.290 × 10^5^ − 3.638 × 10^−3^j	0 4 4	3.645 × 10^5^ − 2.895 × 10^−4^j
–	–	–	–	0 4 356	3.645 × 10^5^ + 8.692 × 10^−4^j
–	–	–	–	0 4 358	1.458 × 10^6^ + 1.344 × 10^−3^j
*k*_1_ *k*_2_ *k*_3_	*C*_2223_	*k*_1_ *k*_2_ *k*_3_	*C*_2233_	*k*_1_ *k*_2_ *k*_3_	*C*_2312_
0 0 0	−3.273 × 10^−4^ + 0j	0 0 0	5.832 × 10^6^ + 0j	0 0 0	−5.371 × 10^−4^ + 0j
0 2 1	6.770 × 10^−5^ − 1.458 × 10^6^j	0 0 2	2.916 × 10^6^ − 8.777 × 10^−4^j	0 2 1	1.458 × 10^6^ − 9.363 × 10^−4^j
0 2 3	1.475 × 10^−3^ + 1.458 × 10^6^j	0 4 0	−2.916 × 10^6^ + 4.489 × 10^−3^j	0 2 3	−1.458 × 10^6^ − 4.469 × 10^−4^j
0 2 357	1.473 × 10^−3^ + 1.458 × 10^6^j	0 4 2	−1.458 × 10^6^ + 3.561 × 10^−3^j	0 2 357	1.458 × 10^6^ − 3.113 × 10^−4^j
0 2 359	−1.745 × 10^−3^ − 1.458 × 10^6^j	0 4 358	−1.458 × 10^6^ + 2.759 × 10^−3^j	0 2 359	−1.458 × 10^6^ + 2.436 × 10^−3^j
0 4 1	−2.036 × 10^−3^ − 2.187 × 10^6^j	–	–	0 4 1	7.290 × 10^5^ − 3.767 × 10^−4^j
0 4 3	4.109 × 10^−4^ − 7.290 × 10^5^j	–	–	0 4 3	7.290 × 10^5^ + 7.465 × 10^−4^j
0 4 357	1.317 × 10^−4^ − 7.290 × 10^5^j	–	–	0 4 357	−7.290 × 10^5^ − 4.772 × 10^−4^j
0 4 359	−1.982 × 10^−3^ − 2.187 × 10^6^j	–	–	0 4 359	−7.290 × 10^5^ + 6.635 × 10^−4^j
*k*_1_ *k*_2_ *k*_3_	*C*_2313_	*k*_1_ *k*_2_ *k*_3_	*C*_2323_	*k*_1_ *k*_2_ *k*_3_	*C*_3312_
0 0 0	−4.698 × 10^−4^ + 0j	0 0 0	1.166 × 10^7^ + 0j	0 0 0	5.534 × 10^−4^ + 0j
0 2 2	2.663 × 10^−3^ + 1.458 × 10^6^j	0 2 0	−2.916 × 10^6^ + 3.338 × 10^−3^j	0 0 2	2.781 × 10^−4^ + 2.916 × 10^6^j
0 2 358	−1.665 × 10^−3^ − 1.458 × 10^6^j	0 2 2	1.458 × 10^6^ + 2.699 × 10^−3^j	0 4 2	−6.452 × 10^−3^ − 1.458 × 10^6^j
0 4 2	−3.239 × 10^−3^ − 1.458 × 10^6^j	0 2 358	1.458 × 10^6^ + 3.103 × 10^−3^j	0 4 358	5.710 × 10^−3^ + 1.458 × 10^6^j
0 4 358	2.674 × 10^−3^ + 1.458 × 10^6^j	0 4 0	−2.916 × 10^6^ + 1.859 × 10^−3^j	–	–
–	–	0 4 2	−1.458 × 10^6^ + 6.743 × 10^−4^j	–	–
–	–	0 4 358	−1.458 × 10^6^ − 9.184 × 10^−4^j	–	–
*k*_1_ *k*_2_ *k*_3_	*C*_3313_	*k*_1_ *k*_2_ *k*_3_	*C*_3323_	*k*_1_ *k*_2_ *k*_3_	*C*_3333_
0 0 0	3.737 × 10^−4^ + 0j	0 0 0	3.272 × 10^−4^ + 0j	0 0 0	3.499 × 10^7^ + 0j
0 4 1	−2.916 × 10^6^ + 1.377 × 10^−3^j	0 4 1	2.203 × 10^−3^ + 2.916 × 10^6^j	0 4 0	5.832 × 10^6^ + 2.951 × 10^−11^j
0 4 359	2.916 × 10^6^ − 2.212 × 10^−3^j	0 4 359	3.092 × 10^−4^ + 2.916 × 10^6^j	–	–

**Table:** ${\tilde{P}}_{k_{1},k_{2},k_{3}}^{\text{B}}$

*k*_1_ *k*_2_ *k*_3_	*C*_1111_	*k*_1_ *k*_2_ *k*_3_	*C*_1112_	*k*_1_ *k*_2_ *k*_3_	*C*_1113_
0 0 0	1.021 × 10^7^ + 0j	0 0 0	−1.346 × 10^−3^ + 0j	0 0 0	−3.474 × 10^−4^ + 0j
0 0 2	−2.916 × 10^6^ + 4.497 × 10^−4^j	0 0 2	1.024 × 10^−3^ + 1.458 × 10^6^j	0 2 1	−1.458 × 10^6^ + 1.752 × 10^−3^j
0 0 4	−2.187 × 10^6^ − 3.121 × 10^−3^j	0 0 4	3.287 × 10^−4^ + 2.187 × 10^6^j	0 2 3	−1.458 × 10^6^ + 9.201 × 10^−4^j
0 2 0	−2.916 × 10^6^ + 4.497 × 10^−4^j	0 2 4	−5.437 × 10^−4^ − 1.458 × 10^6^j	0 2 357	1.458 × 10^6^ − 8.970 × 10^−4^j
0 2 4	1.458 × 10^6^ + 1.246 × 10^−3^j	0 2 356	−3.103 × 10^−4^ + 1.458 × 10^6^j	0 2 359	1.458 × 10^6^ − 1.470 × 10^−3^j
0 2 356	1.458 × 10^6^ + 5.795 × 10^−4^j	0 4 2	−5.819 × 10^−4^ − 7.290 × 10^5^j	0 4 1	−2.187 × 10^6^ + 3.252 × 10^−3^j
0 4 0	−2.187 × 10^6^ − 3.121 × 10^−3^j	0 4 4	1.197 × 10^−3^ + 3.645 × 10^5^j	0 4 3	7.290 × 10^5^ − 7.335 × 10^−4^j
0 4 2	1.458 × 10^6^ + 1.246 × 10^−3^j	0 4 356	3.065 × 10^−4^ − 3.645 × 10^5^j	0 4 357	−7.290 × 10^5^ − 1.229 × 10^−3^j
0 4 4	−3.645 × 10^5^ − 3.397 × 10^−4^j	0 4 358	1.105 × 10^−3^ + 7.290 × 10^5^j	0 4 359	2.187 × 10^6^ − 1.318 × 10^−3^j
0 4 356	−3.645 × 10^5^ + 7.221 × 10^−12^j	–	–	–	–
0 4 358	1.458 × 10^6^ − 5.795 × 10^−4^j	–	–	–	–
*k*_1_ *k*_2_ *k*_3_	*C*_1122_	*k*_1_ *k*_2_ *k*_3_	*C*_1123_	*k*_1_ *k*_2_ *k*_3_	*C*_1133_
0 0 0	1.895 × 10^7^ + 0j	0 0 0	−1.405 × 10^−3^ + 0j	0 0 0	2.916 × 10^7^ + 0j
0 0 4	2.187 × 10^6^ + 2.054 × 10^−3^j	0 2 1	3.890 × 10^−3^ + 4.374 × 10^6^j	0 0 2	−2.916 × 10^6^ + 2.518 × 10^−3^j
0 2 0	−8.748 × 10^6^ − 5.686 × 10^−3^j	0 2 3	1.772 × 10^−3^ + 1.458 × 10^6^j	0 2 0	5.832 × 10^6^ − 5.718 × 10^−4^j
0 2 4	−1.458 × 10^6^ − 1.370 × 10^−4^j	0 2 357	1.828 × 10^−3^ + 1.458 × 10^6^j	0 2 2	2.916 × 10^6^ − 3.981 × 10^−3^j
0 2 356	−1.458 × 10^6^ + 2.013 × 10^−3^j	0 2 359	1.475 × 10^−3^ + 4.374 × 10^6^j	0 2 358	2.916 × 10^6^ + 4.465 × 10^−4^j
0 4 0	−7.290 × 10^5^ − 1.318 × 10^−2^j	0 4 1	1.098 × 10^−3^ + 7.290 × 10^5^j	0 4 0	2.916 × 10^6^ − 5.226 × 10^−4^j
0 4 4	3.645 × 10^5^ + 5.098 × 10^−3^j	0 4 3	3.059 × 10^−3^ − 7.290 × 10^5^j	0 4 2	−1.458 × 10^6^ + 9.562 × 10^−3^j
0 4 356	3.645 × 10^5^ + 4.815 × 10^−3^j	0 4 357	−1.042 × 10^−3^ − 7.290 × 10^5^j	0 4 358	−1.458 × 10^6^ + 7.009 × 10^−3^j
–	–	0 4 359	−8.954 × 10^−4^ + 7.290 × 10^5^j	–	–
*k*_1_ *k*_2_ *k*_3_	*C*_1212_	*k*_1_ *k*_2_ *k*_3_	*C*_1312_	*k*_1_ *k*_2_ *k*_3_	*C*_1313_
0 0 0	−4.374 × 10^6^ + 0j	0 0 0	8.664 × 10^−4^ + 0j	0 0 0	−5.832 × 10^6^ + 0j
0 0 4	2.187 × 10^6^ − 7.102 × 10^−4^j	0 2 1	3.505 × 10^−3^ − 1.458 × 10^6^j	0 0 2	2.916 × 10^6^ − 1.366 × 10^−5^j
0 2 0	2.916 × 10^6^ − 3.869 × 10^−3^j	0 2 3	−2.036 × 10^−3^ + 1.458 × 10^6^j	0 4 0	2.916 × 10^6^ − 3.465 × 10^−3^j
0 2 4	−1.458 × 10^6^ + 2.976 × 10^−3^j	0 2 357	1.196 × 10^−3^ + 1.458 × 10^6^j	0 4 2	−1.458 × 10^6^ + 2.873 × 10^−3^j
0 2 356	−1.458 × 10^6^ + 2.764 × 10^−3^j	0 2 359	−2.458 × 10^−3^ − 1.458 × 10^6^j	0 4 358	−1.458 × 10^6^ + 2.842 × 10^−3^j
0 4 0	−7.290 × 10^5^ + 4.309 × 10^−3^j	0 4 1	−4.336 × 10^−3^ + 7.290 × 10^5^j	–	–
0 4 4	3.645 × 10^5^ − 2.328 × 10^−3^j	0 4 3	3.380 × 10^−3^ − 7.290 × 10^5^j	–	–
0 4 356	3.645 × 10^5^ − 3.997 × 10^−3^j	0 4 357	−2.269 × 10^−4^ − 7.290 × 10^5^j	–	–
–	–	0 4 359	2.354 × 10^−3^ + 7.290 × 10^5^j	–	–
*k*_1_ *k*_2_ *k*_3_	*C*_2212_	*k*_1_ *k*_2_ *k*_3_	*C*_2213_	*k*_1_ *k*_2_ *k*_3_	*C*_2222_
0 0 0	2.227 × 10^−3^ + 0j	0 0 0	1.553 × 10^−3^ + 0j	0 0 0	1.021 × 10^7^ + 0j
0 0 2	8.014 × 10^−4^ + 1.458 × 10^6^j	0 2 1	−4.374 × 10^6^ − 8.811 × 10^−5^j	0 0 2	2.916 × 10^6^ − 2.431 × 10^−3^j
0 0 4	−1.646 × 10^−3^ − 2.187 × 10^6^j	0 2 3	1.458 × 10^6^ − 2.859 × 10^−3^j	0 0 4	−2.187 × 10^6^ + 2.792 × 10^−3^j
0 2 4	1.432 × 10^−3^ + 1.458 × 10^6^j	0 2 357	−1.458 × 10^6^ + 6.060 × 10^−4^j	0 2 0	−2.916 × 10^6^ − 2.517 × 10^−3^j
0 2 356	1.473 × 10^−3^ − 1.458 × 10^6^j	0 2 359	4.374 × 10^6^ + 1.237 × 10^−4^j	0 2 4	1.458 × 10^6^ + 1.090 × 10^−3^j
0 4 2	−1.079 × 10^−4^ − 7.290 × 10^5^j	0 4 1	−7.290 × 10^5^ − 6.559 × 10^−4^j	0 2 356	1.458 × 10^6^ + 1.432 × 10^−3^j
0 4 4	−1.413 × 10^−3^ − 3.645 × 10^5^j	0 4 3	−7.290 × 10^5^ − 3.227 × 10^−3^j	0 4 0	−2.187 × 10^6^ − 5.437 × 10^−4^j
0 4 356	−2.592 × 10^−4^ + 3.645 × 10^5^j	0 4 357	7.290 × 10^5^ + 2.268 × 10^−4^j	0 4 2	−1.458 × 10^6^ + 2.247 × 10^−3^j
0 4 358	4.406 × 10^−4^ + 7.290 × 10^5^j	0 4 359	7.290 × 10^5^ + 3.400 × 10^−3^j	0 4 4	−3.645 × 10^5^ − 1.823 × 10^−3^j
–	–	–	–	0 4 356	−3.645 × 10^5^ + 1.969 × 10^−4^j
–	–	–	–	0 4 358	−1.458 × 10^6^ − 3.514 × 10^−3^j
*k*_1_ *k*_2_ *k*_3_	*C*_2223_	*k*_1_ *k*_2_ *k*_3_	*C*_2233_	*k*_1_ *k*_2_ *k*_3_	*C*_2312_
0 0 0	3.273 × 10^−4^ + 0j	0 0 0	2.916 × 10^7^ + 0j	0 0 0	−1.855 × 10^−3^ + 0j
0 2 1	−6.770 × 10^−5^ + 1.458 × 10^6^j	0 0 2	2.916 × 10^6^ + 2.189 × 10^−3^j	0 2 1	1.458 × 10^6^ − 2.242 × 10^−3^j
0 2 3	−1.475 × 10^−3^ − 1.458 × 10^6^j	0 2 0	5.832 × 10^6^ + 3.827 × 10^−4^j	0 2 3	1.458 × 10^6^ + 9.985 × 10^−4^j
0 2 357	−1.473 × 10^−3^ − 1.458 × 10^6^j	0 2 2	−2.916 × 10^6^ − 8.557 × 10^−4^j	0 2 357	−1.458 × 10^6^ + 9.966 × 10^−4^j
0 2 359	1.745 × 10^−3^ + 1.458 × 10^6^j	0 2 358	−2.916 × 10^6^ + 2.756 × 10^−3^j	0 2 359	−1.458 × 10^6^ − 2.525 × 10^−3^j
0 4 1	2.036 × 10^−3^ + 2.187 × 10^6^j	0 4 0	2.916 × 10^6^ − 1.242 × 10^−3^j	0 4 1	−7.290 × 10^5^ + 9.776 × 10^−4^j
0 4 3	−4.109 × 10^−4^ + 7.290 × 10^5^j	0 4 2	1.458 × 10^6^ − 5.018 × 10^−3^j	0 4 3	−7.290 × 10^5^ − 4.229 × 10^−3^j
0 4 357	−1.317 × 10^−4^ + 7.290 × 10^5^j	0 4 358	1.458 × 10^6^ − 7.378 × 10^−3^j	0 4 357	7.290 × 10^5^ + 1.011 × 10^−4^j
0 4 359	1.982 × 10^−3^ + 2.187 × 10^6^j	–	–	0 4 359	7.290 × 10^5^ + 3.638 × 10^−3^j
*k*_1_ *k*_2_ *k*_3_	*C*_2313_	*k*_1_ *k*_2_ *k*_3_	*C*_2323_	*k*_1_ *k*_2_ *k*_3_	*C*_3312_
0 0 0	−5.534 × 10^−4^ + 0j	0 0 0	−5.832 × 10^6^ + 0j	0 0 0	−1.994 × 10^−3^ + 0j
0 0 2	−2.781 × 10^−4^ − 2.916 × 10^6^j	0 0 2	−2.916 × 10^6^ + 8.777 × 10^−4^j	0 0 2	2.216 × 10^−3^ + 2.916 × 10^6^j
0 4 2	6.452 × 10^−3^ + 1.458 × 10^6^j	0 4 0	2.916 × 10^6^ − 4.489 × 10^−3^j	0 2 2	−3.090 × 10^−3^ − 2.916 × 10^6^j
0 4 358	−5.710 × 10^−3^ − 1.458 × 10^6^j	0 4 2	1.458 × 10^6^ − 3.561 × 10^−3^j	0 2 358	−1.514 × 10^−3^ + 2.916 × 10^6^j
–	–	0 4 358	1.458 × 10^6^ − 2.759 × 10^−3^j	0 4 2	3.003 × 10^−3^ + 1.458 × 10^6^j
–	–	–	–	0 4 358	−1.255 × 10^−4^ − 1.458 × 10^6^j
*k*_1_ *k*_2_ *k*_3_	*C*_3313_	*k*_1_ *k*_2_ *k*_3_	*C*_3323_	*k*_1_ *k*_2_ *k*_3_	*C*_3333_
0 0 0	−3.737 × 10^−4^ + 0j	0 0 0	−3.272 × 10^−4^ + 0j	0 0 0	1.166 × 10^7^ + 0j
0 4 1	2.916 × 10^6^ − 1.377 × 10^−3^j	0 4 1	−2.203 × 10^−3^ − 2.916 × 10^6^j	0 4 0	−5.832 × 10^6^ − 1.367 × 10^−3^j
0 4 359	−2.916 × 10^6^ + 2.212 × 10^−3^j	0 4 359	−3.092 × 10^−4^ − 2.916 × 10^6^j	–	–

**Table:** ${\tilde{P}}_{k_{1},k_{2},k_{3}}^{\text{D}}$

*k*_1_ *k*_2_ *k*_3_	*C*_1111_	*k*_1_ *k*_2_ *k*_3_	*C*_1112_	*k*_1_ *k*_2_ *k*_3_	*C*_1113_
0 0 0	6.561 × 10^6^ + 0j	0 0 0	3.823 × 10^−3^ + 0j	0 0 0	−1.282 × 10^−3^ + 0j
0 0 2	−4.374 × 10^6^ + 1.160 × 10^−3^j	0 0 2	−2.355 × 10^−3^ + 2.187 × 10^6^j	0 2 1	−2.187 × 10^6^ + 1.105 × 10^−3^j
0 0 4	1.093 × 10^6^ − 2.072 × 10^−3^j	0 0 4	1.071 × 10^−3^ − 1.093 × 10^6^j	0 2 3	7.290 × 10^5^ − 2.142 × 10^−3^j
0 2 0	−4.374 × 10^6^ + 1.160 × 10^−3^j	0 2 2	6.666 × 10^−4^ − 1.458 × 10^6^j	0 2 357	−7.290 × 10^5^ − 2.591 × 10^−3^j
0 2 2	2.916 × 10^6^ − 1.262 × 10^−3^j	0 2 4	3.187 × 10^−4^ + 7.290 × 10^5^j	0 2 359	2.187 × 10^6^ + 1.453 × 10^−3^j
0 2 4	−7.290 × 10^5^ + 1.499 × 10^−3^j	0 2 356	−1.668 × 10^−3^ − 7.290 × 10^5^j	0 4 1	1.093 × 10^6^ − 1.071 × 10^−3^j
0 2 356	−7.290 × 10^5^ − 6.593 × 10^−4^j	0 2 358	2.999 × 10^−3^ + 1.458 × 10^6^j	0 4 3	−3.645 × 10^5^ + 2.670 × 10^−3^j
0 2 358	2.916 × 10^6^ − 2.659 × 10^−10^j	0 4 2	−3.242 × 10^−4^ + 3.645 × 10^5^j	0 4 357	3.645 × 10^5^ + 2.440 × 10^−3^j
0 4 0	1.093 × 10^6^ − 2.072 × 10^−3^j	0 4 4	−6.012 × 10^−4^ − 1.823 × 10^5^j	0 4 359	−1.093 × 10^6^ − 1.311 × 10^−3^j
0 4 2	−7.290 × 10^5^ + 1.499 × 10^−3^j	0 4 356	8.978 × 10^−4^ + 1.823 × 10^5^j	–	–
0 4 4	1.823 × 10^5^ + 9.732 × 10^−5^j	0 4 358	−1.676 × 10^−3^ − 3.645 × 10^5^j	–	–
0 4 356	1.823 × 10^5^ + 9.683 × 10^−12^j	–	–	–	–
0 4 358	−7.290 × 10^5^ + 6.593 × 10^−4^j	–	–	–	–
*k*_1_ *k*_2_ *k*_3_	*C*_1122_	*k*_1_ *k*_2_ *k*_3_	*C*_1123_	*k*_1_ *k*_2_ *k*_3_	*C*_1133_
0 0 0	2.187 × 10^6^ + 0j	0 0 0	−2.897 × 10^−4^ + 0j	0 0 0	2.916 × 10^6^ + 0j
0 0 4	−1.094 × 10^6^ + 8.084 × 10^−4^j	0 2 1	−4.025 × 10^−4^ + 7.290 × 10^5^j	0 0 2	−1.458 × 10^6^ − 1.382 × 10^−4^j
0 2 0	−1.458 × 10^6^ + 1.200 × 10^−3^j	0 2 3	2.409 × 10^−4^ − 7.290 × 10^5^j	0 4 0	−1.458 × 10^6^ − 6.165 × 10^−6^j
0 2 4	7.290 × 10^5^ − 1.126 × 10^−3^j	0 2 357	−4.364 × 10^−4^ − 7.290 × 10^5^j	0 4 2	7.290 × 10^5^ + 4.241 × 10^−4^j
0 2 356	7.290 × 10^5^ − 1.900 × 10^−4^j	0 2 359	1.795 × 10^−4^ + 7.290 × 10^5^j	0 4 358	7.290 × 10^5^ + 1.654 × 10^−4^j
0 4 0	3.645 × 10^5^ − 8.533 × 10^−4^j	0 4 1	8.402 × 10^−4^ − 3.645 × 10^5^j	–	–
0 4 4	−1.823 × 10^5^ + 2.970 × 10^−4^j	0 4 3	−7.023 × 10^−4^ + 3.645 × 10^5^j	–	–
0 4 356	−1.823 × 10^5^ + 5.990 × 10^−4^j	0 4 357	−1.036 × 10^−3^ + 3.645 × 10^5^j	–	–
–	–	0 4 359	4.759 × 10^−4^ − 3.645 × 10^5^j	–	–
*k*_1_ *k*_2_ *k*_3_	*C*_1212_	*k*_1_ *k*_2_ *k*_3_	*C*_1312_	*k*_1_ *k*_2_ *k*_3_	*C*_1313_
0 0 0	2.187 × 10^6^ + 0j	0 0 0	−2.897 × 10^−4^ + 0j	0 0 0	2.916 × 10^6^ + 0j
0 0 4	−1.094 × 10^6^ + 8.084 × 10^−4^j	0 2 1	−4.025 × 10^−4^ + 7.290 × 10^5^j	0 0 2	−1.458 × 10^6^ − 1.382 × 10^−4^j
0 2 0	−1.458 × 10^6^ + 1.200 × 10^−3^j	0 2 3	2.409 × 10^−4^ − 7.290 × 10^5^j	0 4 0	−1.458 × 10^6^ − 6.165 × 10^−6^j
0 2 4	7.290 × 10^5^ − 1.126 × 10^−3^j	0 2 357	−4.364 × 10^−4^ − 7.290 × 10^5^j	0 4 2	7.290 × 10^5^ + 4.241 × 10^−4^j
0 2 356	7.290 × 10^5^ − 1.900 × 10^−4^j	0 2 359	1.795 × 10^−4^ + 7.290 × 10^5^j	0 4 358	7.290 × 10^5^ + 1.654 × 10^−4^j
0 4 0	3.645 × 10^5^ − 8.533 × 10^−4^j	0 4 1	8.402 × 10^−4^ − 3.645 × 10^5^j	–	–
0 4 4	−1.823 × 10^5^ + 2.970 × 10^−4^j	0 4 3	−7.023 × 10^−4^ + 3.645 × 10^5^j	–	–
0 4 356	−1.823 × 10^5^ + 5.990 × 10^−4^j	0 4 357	−1.036 × 10^−3^ + 3.645 × 10^5^j	–	–
–	–	0 4 359	4.759 × 10^−4^ − 3.645 × 10^5^j	–	–
*k*_1_ *k*_2_ *k*_3_	*C*_2212_	*k*_1_ *k*_2_ *k*_3_	*C*_2213_	*k*_1_ *k*_2_ *k*_3_	*C*_2222_
0 0 0	2.792 × 10^−3^ + 0j	0 0 0	3.074 × 10^−5^ + 0j	0 0 0	6.561 × 10^6^ + 0j
0 0 2	2.951 × 10^−3^ + 2.187 × 10^6^j	0 2 1	−7.290 × 10^5^ + 2.330 × 10^−4^j	0 0 2	4.374 × 10^6^ + 7.014 × 10^−4^j
0 0 4	2.232 × 10^−3^ + 1.093 × 10^6^j	0 2 3	−7.290 × 10^5^ + 5.117 × 10^−4^j	0 0 4	1.093 × 10^6^ + 1.030 × 10^−3^j
0 2 2	−3.103 × 10^−3^ − 1.458 × 10^6^j	0 2 357	7.290 × 10^5^ + 5.010 × 10^−4^j	0 2 0	−4.374 × 10^6^ + 9.433 × 10^−4^j
0 2 4	−2.957 × 10^−3^ − 7.290 × 10^5^j	0 2 359	7.290 × 10^5^ + 5.058 × 10^−4^j	0 2 2	−2.916 × 10^6^ + 1.423 × 10^−4^j
0 2 356	−5.481 × 10^−4^ + 7.290 × 10^5^j	0 4 1	3.645 × 10^5^ − 1.666 × 10^−4^j	0 2 4	−7.290 × 10^5^ − 1.449 × 10^−4^j
0 2 358	−1.477 × 10^−3^ + 1.458 × 10^6^j	0 4 3	3.645 × 10^5^ + 1.777 × 10^−4^j	0 2 356	−7.290 × 10^5^ + 8.615 × 10^−4^j
0 4 2	1.298 × 10^−3^ + 3.645 × 10^5^j	0 4 357	−3.645 × 10^5^ − 3.247 × 10^−4^j	0 2 358	−2.916 × 10^6^ + 1.174 × 10^−3^j
0 4 4	1.733 × 10^−3^ + 1.823 × 10^5^j	0 4 359	−3.645 × 10^5^ − 1.046 × 10^−3^j	0 4 0	1.093 × 10^6^ − 2.872 × 10^−3^j
0 4 356	2.726 × 10^−4^ − 1.823 × 10^5^j	–	–	0 4 2	7.290 × 10^5^ − 1.485 × 10^−3^j
0 4 358	9.600 × 10^−4^ − 3.645 × 10^5^j	–	–	0 4 4	1.823 × 10^5^ − 3.610 × 10^−4^j
–	–	–	–	0 4 356	1.823 × 10^5^ + 5.363 × 10^−4^j
–	–	–	–	0 4 358	7.290 × 10^5^ − 2.104 × 10^−3^j
*k*_1_ *k*_2_ *k*_3_	*C*_2223_	*k*_1_ *k*_2_ *k*_3_	*C*_2233_	*k*_1_ *k*_2_ *k*_3_	*C*_2312_
0 0 0	1.254 × 10^−3^ + 0j	0 0 0	2.916 × 10^6^ + 0j	0 0 0	3.074 × 10^−5^ + 0j
0 2 1	−1.485 × 10^−3^ + 2.187 × 10^6^j	0 0 2	1.458 × 10^6^ − 4.806 × 10^−4^j	0 2 1	−7.290 × 10^5^ + 2.330 × 10^−4^j
0 2 3	−2.290 × 10^−3^ + 7.290 × 10^5^j	0 4 0	−1.458 × 10^6^ + 1.795 × 10^−4^j	0 2 3	−7.290 × 10^5^ + 5.117 × 10^−4^j
0 2 357	1.152 × 10^−3^ + 7.290 × 10^5^j	0 4 2	−7.290 × 10^5^ + 2.234 × 10^−4^j	0 2 357	7.290 × 10^5^ + 5.010 × 10^−4^j
0 2 359	3.268 × 10^−4^ + 2.187 × 10^6^j	0 4 358	−7.290 × 10^5^ − 4.228 × 10^−4^j	0 2 359	7.290 × 10^5^ + 5.058 × 10^−4^j
0 4 1	6.655 × 10^−4^ − 1.093 × 10^6^j	–	–	0 4 1	3.645 × 10^5^ − 1.666 × 10^−4^j
0 4 3	1.517 × 10^−3^ − 3.645 × 10^5^j	–	–	0 4 3	3.645 × 10^5^ + 1.777 × 10^−4^j
0 4 357	−1.949 × 10^−3^ − 3.645 × 10^5^j	–	–	0 4 357	−3.645 × 10^5^ − 3.247 × 10^−4^j
0 4 359	−8.841 × 10^−4^ − 1.093 × 10^6^j	–	–	0 4 359	−3.645 × 10^5^ − 1.046 × 10^−3^j
*k*_1_ *k*_2_ *k*_3_	*C*_2313_	*k*_1_ *k*_2_ *k*_3_	*C*_2323_	*k*_1_ *k*_2_ *k*_3_	*C*_3312_
0 0 0	−1.640 × 10^−3^ + 0j	0 0 0	2.916 × 10^6^ + 0j	0 0 0	−1.640 × 10^−3^ + 0j
0 0 2	1.228 × 10^−3^ + 1.458 × 10^6^j	0 0 2	1.458 × 10^6^ − 4.806 × 10^−4^j	0 0 2	1.228 × 10^−3^ + 1.458 × 10^6^j
0 4 2	−1.516 × 10^−3^ − 7.290 × 10^5^j	0 4 0	−1.458 × 10^6^ + 1.795 × 10^−4^j	0 4 2	−1.516 × 10^−3^ − 7.290 × 10^5^j
0 4 358	−4.549 × 10^−5^ + 7.290 × 10^5^j	0 4 2	−7.290 × 10^5^ + 2.234 × 10^−4^j	0 4 358	−4.549 × 10^−5^ + 7.290 × 10^5^j
–	–	0 4 358	−7.290 × 10^5^ − 4.228 × 10^−4^j	–	–
*k*_1_ *k*_2_ *k*_3_	*C*_3313_	*k*_1_ *k*_2_ *k*_3_	*C*_3323_	*k*_1_ *k*_2_ *k*_3_	*C*_3333_
0 0 0	1.518 × 10^−3^ + 0j	0 0 0	5.058 × 10^−4^ + 0j	0 0 0	1.750 × 10^7^ + 0j
0 2 1	−2.916 × 10^6^ + 1.574 × 10^−3^j	0 2 1	1.420 × 10^−3^ + 2.916 × 10^6^j	0 2 0	1.166 × 10^7^ + 1.264 × 10^−1^j
0 2 359	2.916 × 10^6^ − 2.529 × 10^−3^j	0 2 359	1.340 × 10^−3^ + 2.916 × 10^6^j	0 4 0	2.916 × 10^6^ + 5.968 × 10^−2^j
0 4 1	−1.458 × 10^6^ − 1.493 × 10^−3^j	0 4 1	−9.979 × 10^−4^ + 1.458 × 10^6^j	–	–
0 4 359	1.458 × 10^6^ + 2.746 × 10^−4^j	0 4 359	−1.115 × 10^−3^ + 1.458 × 10^6^j	–	–
